# Clinical impact of acetabular defect severity on hip center of rotation restoration in revision total hip arthroplasty

**DOI:** 10.1016/j.jor.2026.01.015

**Published:** 2026-01-29

**Authors:** Victor Rafael Casas Gállego, Miguel Angel Ortega Núñez, Basilio José de la Torre Escuredo

**Affiliations:** a Department of Surgery, Medical and Social Sciences, Faculty of Medicine and Health Sciences, Ramón y Cajal Institute of Sanitary Research (IRYCIS), University of Alcala, Alcala de Henares, 28034, Madrid, Spain; b Department of Orthopedic Surgery, University Hospital Ramón y Cajal, 28034, Madrid, Spain; c Department of Medicine and Medical Specialities, Faculty of Medicine and Health Sciences (CIBER-EHD), Ramón y Cajal Institute of Sanitary Research (IRYCIS), University of Alcalá, Alcalá de Henares, 28034, Madrid, Spain

## Abstract

**Background:**

Acetabular revision is a challenging procedure, especially in patients with large defects. This study aimed to evaluate how the severity of acetabular bone loss influences reconstruction of the center of rotation (COR) and how the COR influences clinical outcomes and patient-reported outcome measures (PROMs).

**Methods:**

Patients who underwent acetabular revision at a tertiary hospital from January 2013 to December 2018 were included. Patients were grouped according to the Paprosky and Saleh classifications. To determine the COR, we applied the method described by Fessy. The Harris Hip Score (HHS), Western Ontario McMaster Arthritis Index (WOMAC) and Short Form 12 (SF-12) were used to determine clinical outcomes and PROMs at a median of 41 months of follow-up. A total of 117 acetabular revisions were performed. The Paprosky classification was I for 54 acetabular defects (46.15 %); II for 36 acetabular defects (30.76 %); and III for 27 acetabular defects (23.07 %). The Saleh classification was as follows: I for 54 (46.15 %); II for 26 (22.22 %); III for 19 (16.23 %); IV for 16 (13.67 %) and V for 2 (1,7 %) acetabular defects.

**Results:**

The percentage of patients who achieved an appropriate COR was 60.1 % according to the Fessy method. This method showed a statistically significant association between the severity of the acetabular defect and the ability to accurately reconstruct the center of rotation. Similarly, no differences were observed in patients’ functional outcomes (HHS, WOMAC, and SF-12 scores) based on whether the hip center of rotation was restored. Likewise, no differences were found in complication rates regardless of COR restoration or the degree of bone loss

**Conclusion:**

These findings suggest that, in complex acetabular revisions, prioritizing stable fixation and bone preservation rather than perfect anatomic restoration of the center of rotation does not adversely affect functional outcomes.

## 1 Introduction

Revision hip arthroplasty has become a common procedure in orthopedic surgery services, mainly because of the increase in primary surgeries performed in recent years as well as the increase in patient life expectancies.^1^

The causes leading to revision total hip arthroplasty include periprosthetic osteolysis and subsequent aseptic loosening of the implant, recurrent dislocation, infection, and polyethylene wear.^2^^,^^3^ Within revision THA surgery, revision of the acetabular component is particularly challenging due to acetabular bone defects, especially in cases of severe bone loss.

The goals of acetabular revision surgery are three-fold: to achieve a reconstruction with an adequate center of rotation (COR) to allow proper biomechanical functioning; to achieve correct fixation of the implants; and finally, to the greatest extent possible, to reestablish the integrity of the bone remnant.^4^^,^^5^ Achieving these goals represents a great challenge for surgeons. Specifically, the presence of severe bone defects or, in certain circumstances, pelvic discontinuity, means that this principle is not always achieved. In this setting, the cup position must achieve a compromise between stable fixation and suitable restoration of biomechanical parameters. Elevation of the hip COR may occur, which could lead to impaired biomechanics, particularly insufficiency of the abductor muscles, altered gait with limping, and increased risk of dislocation and other complications.^6^^,^^7^

On the other hand, patients undergoing this type of surgery often present with high surgical risks due to their advanced age, significant medical comorbidities, and limited capacity for postoperative recovery.^8^^,^^9^ As a result, rather than prioritizing restoration of the anatomical hip center of rotation, surgical efforts are often directed toward optimizing implant stability, even at the expense of restoring normal hip biomechanics and function.

Multiple studies have evaluated predictors of patient-reported outcome measures (PROMs) and clinical outcomes in primary THA^10^, ^11^, ^12^, ^13^, but relatively few studies have assessed PROMs in patients undergoing total hip revision.^2^^,^^14^ Moreover, the indications and treatments for total hip revision differ and may be associated with different results.

The aim of this study was to evaluate how the severity of acetabular bone loss influences the reconstruction of the COR, and how the COR in turn influences clinical outcomes and PROMs. Specifically, we evaluated the acetabular reconstruction of the COR via Fessy method; overall complication rates according to the severity of acetabular bone loss; and whether reconstruction of the COR was associated with clinical outcomes. In this way, understanding the factors that predict patient outcomes may assist surgeons in discussing clinical expectations with patients undergoing acetabular revision.

## 2 Methods

### 2.1 Study design

An analytical observational study was carried out with data from patients who underwent revision THA. The study was approved by the Research Ethics Committee (CEIM registration number: 282/20). All patients were informed about the purpose of the study and provided informed consent for participation.

### 2.2 Patients

**Fig. 1:**
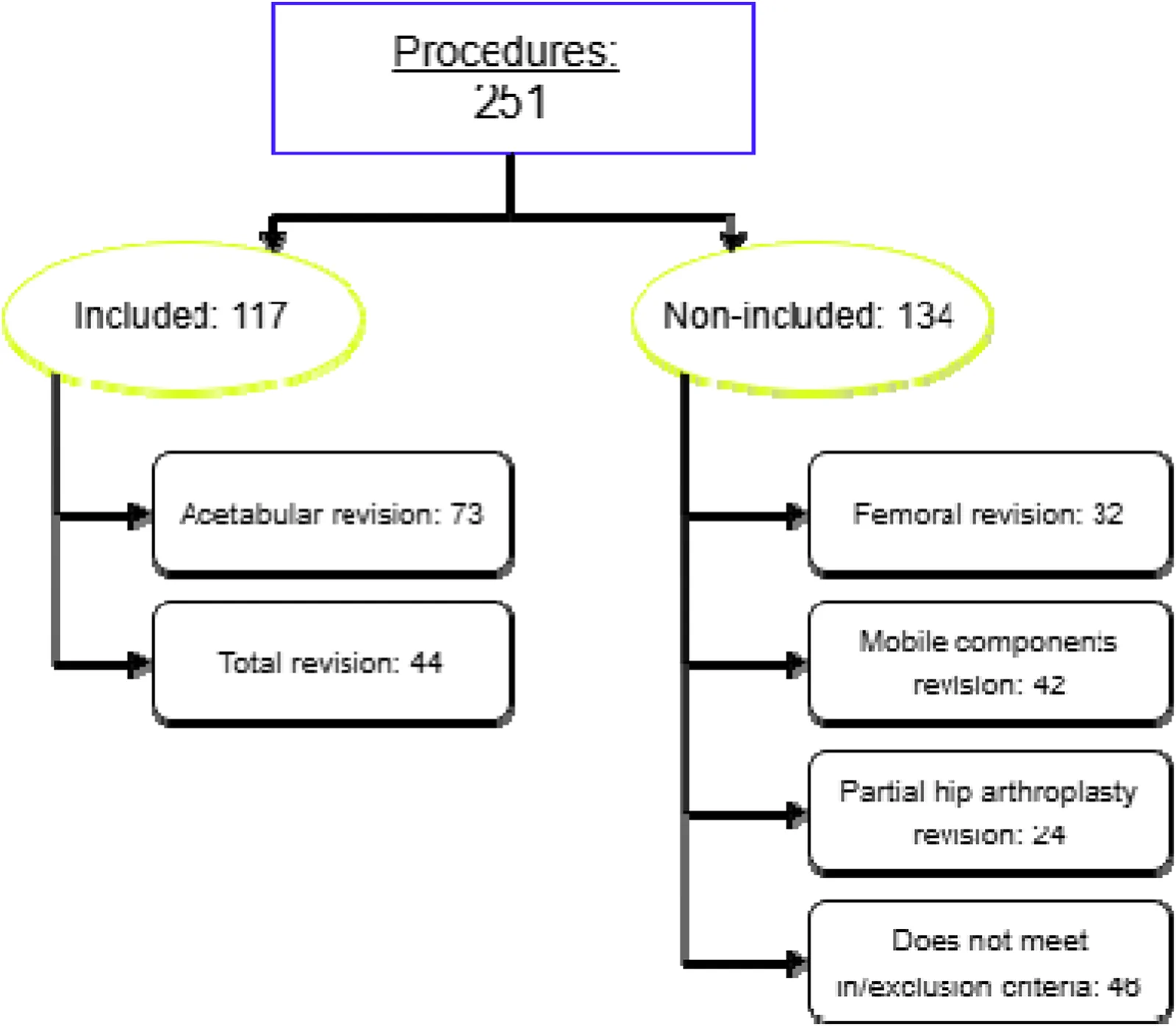
Algorithm of patients included in the study.

Patients who underwent revision THA between January 2013 and December 2018 were included. The study included a total of 251 procedures in 243 patients; of these, 117 procedures in 114 patients involved revision of the acetabular component (Fig. 1). Cases were collected during 2021, and the median follow-up was 41 months.

The exclusion criteria were age below 18 years; revision performed exclusively for the femoral component; surgery primarily due to cancer; implant replacement due to polyethylene wear without extraction of the acetabular component; osteosynthesis failure in the femur; and refusal to participate in the study.

The data collected from the patients included age, sex, body mass index (BMI), diabetes mellitus status, anesthetic risk according to the American Society of Anesthesiologists (ASA) scale, reason for revision surgery, and type of acetabular defect according to the Paprosky and Saleh classifications. Similarly, complications such as prosthetic dislocation, implant mobilization, and peri-implant fractures were analyzed. All the data were collected by one of the authors during the clinical interview.

Defects were divided into mild defects and severe defects. According to Paprosky's classification, mild defects are those defects whose reconstruction involve an intact acetabular tear drop (Paprosky type I to IIB), and severe defects include those with involvement of the tear drop, acetabular fundus, and posterolateral region (Paprosky IIC to IIIB). According to Saleh, mild defects include contained defects presenting with an intact acetabular fundus and columns (Saleh I-II), and severe defects include uncontained acetabular defects (Saleh III - V).

### 2.3 Radiographic evaluation

Anteroposterior (AP) radiographs of the pelvis and axial hip radiography were analyzed to quantify the acetabular defect prior to revision surgery. Similarly, the final postoperative radiographs were evaluated to assess the acetabular reconstruction according to the COR of the hip as well as the presence of radiolucent lines.

The Fessy method were used to evaluate the COR.^15^ According to the Fessy measurement method, an adequate range for the COR was 20 mm–47 mm on the X axis and 9 mm–26 mm on the Y axis.^15^^,^^16^ The data were assessed by two of the authors; no discrepancies greater than 5 mm were detected in any of the measurements.

The presence of radiolucent lines indicative of implant loosening, as well as implant migration, was assessed on the final radiograph 17, 18, 19.

### 2.4 Questionnaires

Three questionnaires were administered. One of them, the Harris Hip Score (HHS) questionnaire was used to evaluate patient's functional capacity.^20^ The other two questionnaires—the Western Ontario and McMaster Universities Arthritis Index (WOMAC)^21^^,^^22^ and the 12-Item Short Form Health Survey (SF-12)^23^ —were used to assess PROMs. All questionnaires were completed by the patients under the supervision of a member of the research team (VC).

### 2.5 Statistical analysis

Statistical analysis was performed with Stata/MP version 17.0. The results are presented as the means, medians and standard deviations (SDs). Student's *t*-test was used to compare independent variables with a parametric distribution between groups to assess the influence of the COR on the results of the questionnaires. A p value < 0.05 was considered to indicate statistical significance.

## 3 Results

### 3.1 Patient demographics

Table 1 presents the descriptive variables of the 117 patients included in our sample.

**Table 1:** Analysis of Patient demographics. The results are presented as means ± standard deviations (SDs), or as totals and percentages. Data on age, weight (kg), body mass index (BMI), sex (female), laterality (right), WOMAC scale score, Harris Hip Score, and SF-12 questionnaire score are included. Comparisons of age, weight, BMI, WOMAC score, Harris Hip Score, and SF-12 score were performed using Student's t-test. Comparisons of sex (female) and laterality (right) were analyzed using the Chi-square test. For ASA score, reason for revision surgery, acetabular defect according to Saleh, dislocation, reoperation, radiolucent lines, and implant mobilization, comparisons were made using Fisher's exact test.

**Patient demographics**				

	*Mild defect (SD/%)*	*Severe defect (SD/%)*	*Total (SD/%)*	*p - Value*

Age	− 75.4 (14.2)	− 71.9 (13.2)	− 74.5 (13.9)	p = 0.196
Weight	− 72.4 (15.2)	− 69.2 (13.9)	− 71.74 (14.8)	p = 0.370
BMI	− 27.1 (5.5)	− 28.3 (4.6)	− 27.44 (5.4)	p = 0.458
Female sex, n (%)	− 44 (38,3 %)	− 21 (18,3 %)	− 65 (55,6 %)	p = 0.783
Right laterality, n (%)	− 45 (38,5 %)	− 19 (16,2 %)	− 64 (54.7 %)	p = 0.695
ASA grade, n (%)	− I: 7 (6.0 %) − II:50 (42.7 %) − III:26 (22.2 %) − IV:1(0.9 %)	− I: 2 (1.7 %) − II:23 (19.7 %) − III: 7 (6.0 %) − IV: 1 (0.9 %)	− I: 9 (7.7 %) − II: 73 (62.4 %) − III: 33 (28.2 %) − IV: 2 (1.8 %)	p = 0.501
Reason for revision surgery, n (%)	− Aseptic loosening:30 (25.6 %) − Peri-implant infection:11 (9,4 %) − Peri-implant dislocation: 16(13.7 %) − Other:27 (23.1 %)	− Aseptic loosening:23 (19.7 %) − Peri-implant infection:5 (4.3 %) − Peri-implant dislocation:0 (0 %) − Other:5 (4.3 %)	− Aseptic loosening:53 (45.3 %) − Peri-implant infection:16 (13.7 %) − Peri-implant dislocation:16 (13.7 %) − Other:32 (27.4 %)	p = 0.001
Acetabular defect Paprosky	− I:54 (46.2 %) − IIA:15 (12.8 %) − IIB:17 (14.6 %)	− IIC:4 (3.4 %) − IIIA: 19 (16.2 %) − IIIB: 8 (6.8 %)		
Acetabular defect Saleh	− I: 54 (46.2 %) − II: 26 (22.2 %)	− III: 19 (16.2 %) − IV: 16 (13.6 %) − V: 2 (1.7 %)		

*Functional assessment scales*				

WOMAC	− Total: 37.0 ± 19.8 − Pain: 5.93 ± 4.24 − Rigidity: 2.76 ± 1.87 − Functional limitation: 28.3 ± 14.9	− Total: 32.4 ± 22.7 − Pain:5.88 ± 5.86 − Rigidity: 1.94 ± 1.91 − Functional limitation: 24.6 ± 16.2	− Total: 35.81 ± 20.52 − Pain: 5.91 ± 4.67 − Rigidity: 2.54 ± 1.90 − Functional limitation: 27.36 ± 15.16	− Total: p = 0.599 − Pain: p = 0.519 − Rigidity: p = 0.429 − Functional limitation: p = 0.540
HHS	− 0.75 ± 0.19	− 0.77 ± 0.2	− 0.75 ± 0.19	p = 0.743
SF-12	− Physical:32.6 ± 11.0 − Mental: 49.7 ± 11.5	− Physical:35.3 ± 12.4 − Mental: 44.1 ± 14.5	− Physical: 33.28 ± 11.36 − Mental: 48.20 ± 12.49	− Physical: p = 0.526 − Mental: p = 0.029

*Complications*				

Dislocation	− No:76 (65.0 %) − Yes (early):6 (5.1 %) − Yes (late):2 (1.7 %)	− No:28 (23.9 %) − Yes(early): 4 (3.4 %) − Yes (late): 1 (0.9 %)	− No: 104 (88.9 %) − Yes (early):10 (8.6 %) − Yes(late):3 (2.5 %)	p = 0.569
Radiolucent lines	− No:76 (65.0 %) − Yes:8 (6.8 %)	− No:29 (24.8 %) − Yes: 4 (3.4 %)	− No:105 (89.7 %) − Yes: 12 (10.3 %)	p = 0.738
Implant mobilization	− No:81 (69.2 %) − Yes: 3 (2.6 %)	− No: 33 (28.2 %) − Yes: 0 (0 %)	− No: 114 (97.4 %) − Yes: 3 (2.6 %)	p = 0.558

### 3.2 Radiological assessment of acetabular reconstruction

**Fig. 2:**
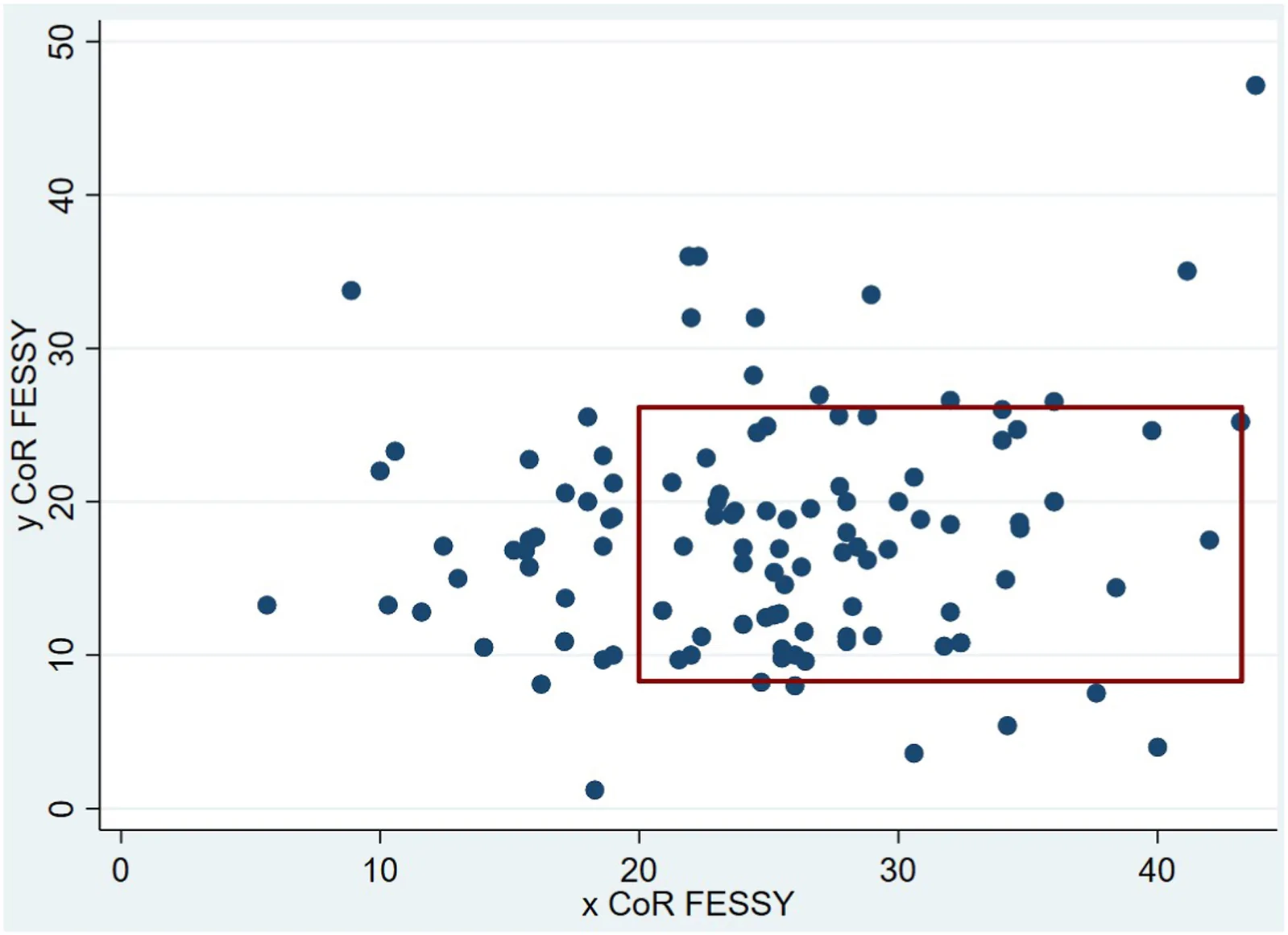
Scatter plot showing the location of the COR on the X and Y axes. The rectangle indicates the safe range according to the Fessy method, spanning from 20 to 47 mm on the X-axis and from 9 to 27 mm on the Y-axis. Each point represents the location of an individual hip, illustrating which hips fall inside and outside the safe range.

When assessing the center of rotation (COR) positioning, the Fessy measurement method identified the COR within the appropriate range in 71 acetabular revision cases (60.1 %) and outside the range in 46 cases (39.9 %). (Fig. 2).

**Fig. 3:**
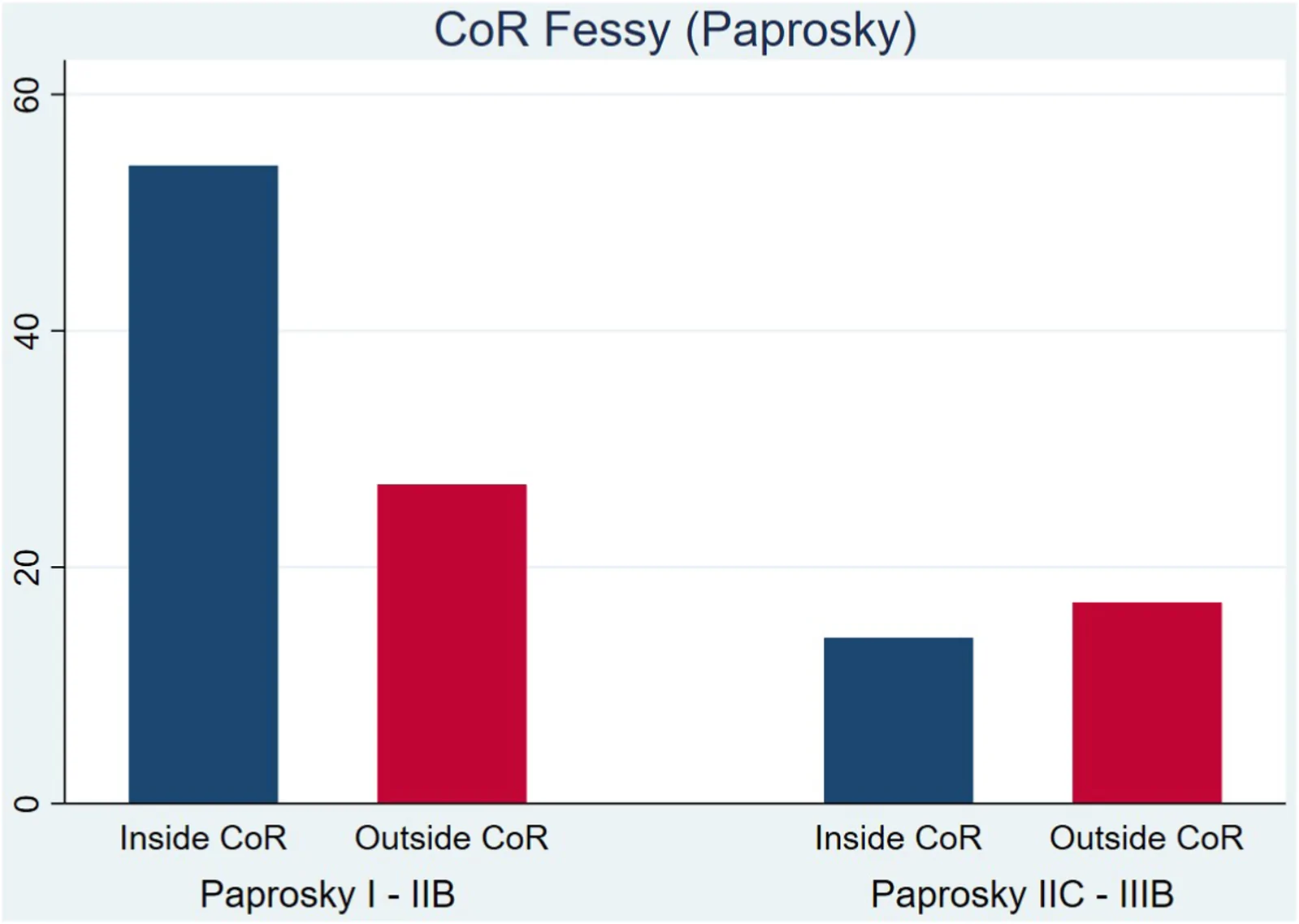
Bar charts. Panel A shows the frequency of findings within and outside the CoR according to the Fessy method, depending on the Paprosky acetabular defect classification.

However, when evaluating the influence of acetabular defect severity on achieving a correct COR, the Fessy method revealed significant differences. We found a statistically significant association between COR accuracy and the Paprosky classification (p = 0.03): a correct COR was achieved in 55 patients (66.7 %) with mild defects but only in 14 patients (45.1 %) with severe defects. A similar trend was observed with the Saleh classification, with values approaching significance (p = 0.058): 54 patients (66.2 %) with mild defects versus 15 patients (46.9 %) with severe defects achieved a COR within the appropriate range after reconstruction (Fig. 3).

These findings indicate that larger acetabular defects make it more challenging to restore an anatomically accurate center of rotation, and they support the sensitivity of the Fessy method in detecting the impact of defect severity on COR reconstruction.

We also investigated whether the presence of radiolucent lines was related to placement of the COR within the appropriate range. Among patients with radiolucent lines at the implant–bone interface, 7 (10.1 %) had CORs within the appropriate range and 5 (11.4 %) had CORs outside it. This difference was not statistically significant.

The associations between the location of the COR and mobilization of the implant were also not significant. We identified 2 patients (2.3 %) with implant mobilization whose CORs were within the appropriate range, and 1 patient (1.1 %) with implant mobilization whose COR was outside the appropriate range. Therefore, no relationship appears to exist between the location of the COR and medium-term mechanical loosening.

The presence of prosthetic instability was also assessed with respect to the location of the COR, and no relevant associations were found. Among patients who experienced instability, 7 (10.1 %) had CORs within the appropriate range and 6 (13.7 %) had CORs outside it.

### 3.3 Functional results

**Fig. 4:**
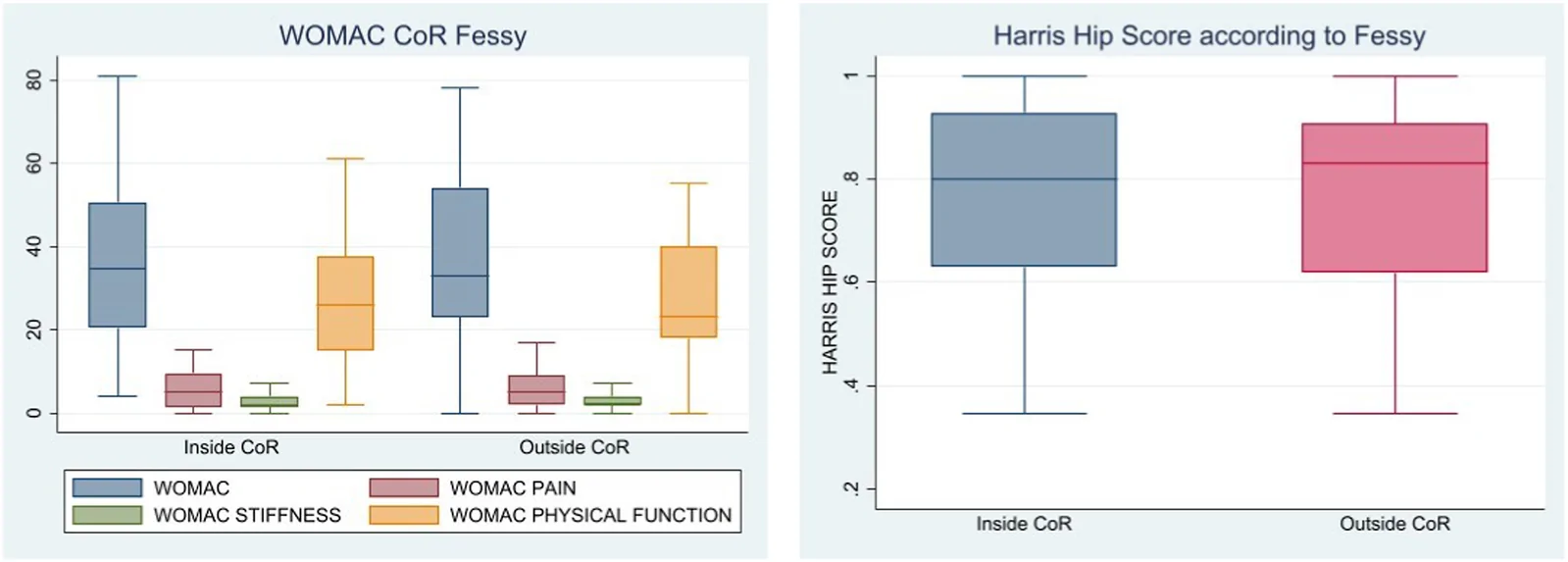
Panel A show the WOMAC index for patients with different Fessy COR locations; the differences between the groups were not significant. Panels B show the results for the Harris Hip Scale.

Differences in functional outcomes were assessed according to COR location using the various functional assessment scales. Regarding the Harris Hip Score, the mean values for patients whose COR was within the appropriate range were 0.75 ± 0.19, compared with 0.76 ± 0.19 in those with CORs outside the appropriate range. Therefore, no differences were observed in Harris Hip Score results between patients with CORs within or outside the appropriate range (Fig. 4 and Table 2).

**Table 2:** Comparison of the Harris Hip Score, WOMAC and Sf-12 functional assessment results for patients whose COR was or was not within the appropriate range. P values were obtained with Student's *t*-test.

	COR within *proper range* *Mean (SD)*	COR outside proper range *Mean (SD)*	*Total*	*P value*
*Harris Hip Score*	0.75 (0.03)	0.76 (0.03)	0.75 (0.02)	p = 0.86
*WOMAC total*	34.52 (3.27)	37.68 (4.40)	35.81 (2.63)	p = 0.55
*SF-12 physical*	34.58 (2.11)	31.41 (1.80)	33.28 (1.45)	p = 0.28
*SF-12 mental*	48.58 (2.09)	47.65 (2.51)	48.20 (1.59)	p = 0.77

With respect to the questionnaires that analyse PROMs, the mean values of the WOMAC questionnaire for patients whose COR was within the appropriate range was 34.52 ± 19.62, while that of patients whose COR was outside the appropriate range was 37.68 ± 22.02. Although patients with CORs outside the appropriate range had clinically worse WOMAC, the differences were not statistically significant (Fig. 4 and Table 2).

**Fig. 5:**
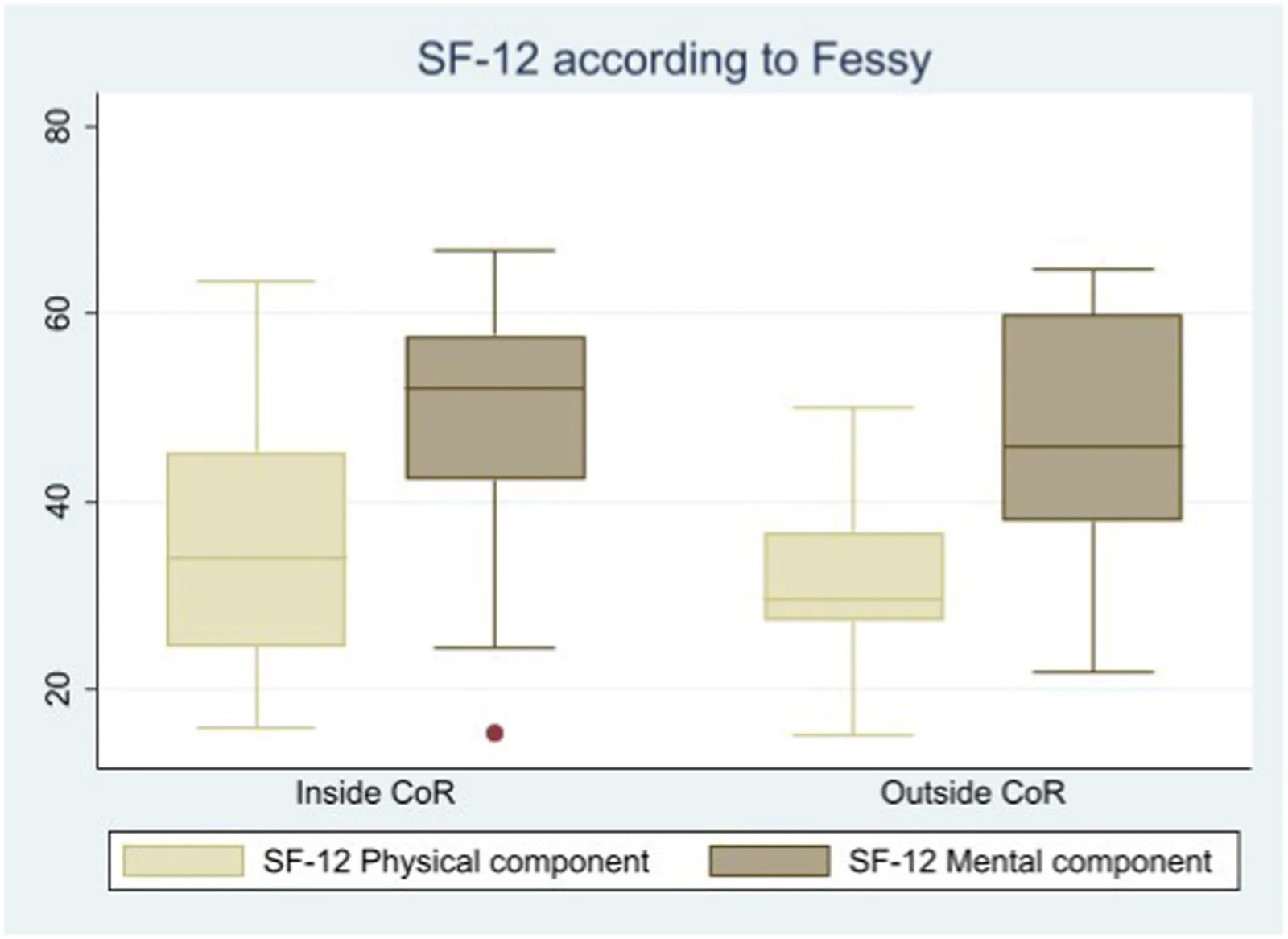
Panels A show the SF-12 score for patients with different Fessy CoR locations. The differences between groups were not significant.

Finally, with respect to the SF-12 global assessment scale for quality of life, no significant differences were found. Notably, although the difference was not significant, patients whose COR was within the appropriate range (34.58 ± 12.71) were higher than in those whose CORs were outside the appropriate range (31.41 ± 9.0) (p = 0.28) (Fig. 5 and Table 2).

## 4 Discussion

This was a single-center study based on a review of 117 acetabular repairs performed in a tertiary hospital. One of the major challenges in acetabular revision is reconstruction of the acetabulum at the precise anatomic center of the hip joint, especially in patients with major bone loss. Different studies have shown that placing the center of rotation in an elevated position increases polyethylene wear in clinical practice.^24^ The same has been demonstrated in studies conducted by one of the authors using finite elements when the COR is elevated.^25^^,^^26^ From a clinical perspective, anatomical reconstruction entails a better distribution of forces in the hip joint, resulting in better biomechanical functioning of the joint 27, 28, 29. Therefore, most authors recommend correct establishment of the COR.

Nevertheless, few studies have evaluated how acetabular bone loss severity influences placement of the COR during reconstruction and how the COR in turn influences clinical outcomes and PROMs.

We used the Fessy method to evaluate whether the center of rotation (COR) was restored to an appropriate position, as COR assessment methods that reference the radiographic teardrop have been shown to provide a more precise determination of the anatomical hip center. This has also been supported by Schofer et al.,^15^ who reported that teardrop-based measurements allow accurate localization of the hip joint center and are robust against radiographic magnification variability. Using the Fessy method, we found that 60.1 % of patients achieved a COR within the ideal range.

Furthermore, we observed that the likelihood of achieving an appropriate COR was associated with the severity of the acetabular defect. A statistically significant association was found between COR accuracy and the Paprosky classification (p = 0.03): a correct COR was obtained in 55 patients (66.7 %) with mild defects but only in 14 patients (45.1 %) with severe defects. These findings indicate that severe acetabular defects present greater technical challenges and make accurate restoration of the COR more difficult during reconstruction.

We found that the likelihood of achieving an appropriate COR according to the Fessy method during reconstruction was associated with the severity of the acetabular defect. In contrast, this association was not observed when COR was assessed using the Ranawat method, suggesting that the Fessy method may be more sensitive in detecting the biomechanical implications of advanced bone loss. This may be due to the fact that measurements referencing the position of the radiographic teardrop sign are more precise for evaluating the anatomical COR, as has also been reported by other authors.^15^

However, the differences in the percentages of patients with and without appropriate COR locations were not significant for among those with radiolucent lines or implant mobility. This may be because the definition of the ‘appropriate range’ is very narrow. In practical terms, our results suggest that moderate deviations from the ideal COR do not appear to negatively affect these outcomes. This finding differs from what has traditionally been reported in the literature, where placing the hip outside the ideal center of rotation has been associated with higher rates of implant failure, especially in primary surgery for hip dysplasia 30, 31, 32. However, more recent studies have not observed this finding.^33^

Overall, our results indicate that factors other than precise COR restoration may play a more important role in clinical success during acetabular revision. For this reason, we believe that achieving stable fixation to the greatest possible amount of host bone is more important, as long as the COR remains within the general limits proposed by Fessy.

Regarding instability, we did not find a significant effect of COR location, although the dislocation rate was slightly higher in patients whose CORs were outside the ideal range. It should be noted that we did not include patients with a substantial elevation of the COR and that for most patients whose COR was outside the acceptable range, the COR was medialized, which can actually increase joint stability.^34^

Another significant finding was that clinical outcomes and PROMs in acetabular revision surgery were not affected by the classification of the location of the COR. Although elevation of the COR alters the biomechanical functioning of the hip joint^28^ and can affect the clinical outcomes of the patient postoperatively, the literature is contradictory in this regard. In primary hip surgery, Traina et al. evaluated 44 patients who underwent primary hip arthroplasty for hip dysplasia with a high COR prior to surgery. They did not find differences in functional results between patients with a high COR and those without.^29^ Few studies have evaluated the effect of COR location on clinical outcomes and PROMs in revision surgery, however. Epinette et al. analyzed functional capacity at 10 years of age in patients with a diagnosis of severe protrusion following acetabular revision surgery and reported no significant differences between patients who achieved a high COR and those who did not.^33^ Therefore, we consider correct stability of the components more essential for achieving adequate osseointegration of the components than attempting to restore the COR anatomically.

Similarly, the analysis of the PROMs via the WOMAC and SF-12 questionnaires did not reveal striking changes between the location of the COR.

Our study has several limitations. First, from a functional perspective, the results prior to surgery were not compared with the postoperative results. However, we only wanted to consider the functional status of the patient to compare the results perceived by the patient. Second, our work includes a relatively short follow-up time (41 months). We believe that this is an appropriate duration for assessing the results of revision surgery, given the results of other studies^12^^,^^35^^,^^36^ and because the average age and medical comorbidities of these patients make appropriate follow-up difficult. Finally, the lack of significant differences with respect to reconstruction of the COR may be due to the narrow margin studied when CORs reconstructed in elevated positions are taken into account. However, we believe that this study has some relevance, since we were unable to identify studies that compared clinical and functional results between patients who did or did not achieve an adequate COR in acetabular revision surgery.

## 5 Conclusion

In conclusion, this single-center study of 117 patients undergoing acetabular revision demonstrated that the ability to restore the center of rotation (COR) was associated with the severity of acetabular bone loss. However, moderate deviations in COR positioning were not associated with differences in clinical outcomes or PROMs. These findings may assist surgeons in setting appropriate expectations with patients undergoing acetabular revision, particularly when reconstruction of the COR is required. Further research is warranted to evaluate the clinical impact of positioning the COR at a markedly superior location.

## Consent for publication

Not applicable. This manuscript does not include any identifying images or personal or clinical details of individual participants.

## Availability of data and materials

The data supporting the findings of this study are available from the corresponding author upon reasonable request.

## Ethics approval and consent to participate

The study was approved by the Research Ethics Committee with Medicines (Comité de Ética de la Investigación con Medicamentos) of the Instituto Ramón y Cajal de Investigación Sanitaria (IRYCIS), Hospital Universitario Ramón y Cajal, Madrid, Spain (approval code 282/20).

The study was conducted in accordance with the Declaration of Helsinki.

Written informed consent was obtained from all participants prior to their inclusion in the study.

## Author contributions

**Victor Rafael Casas Gállego, MD, PhD:** Conceptualization, study design, data acquisition, data interpretation, writing – original draft, and corresponding author responsibilities.

**Miguel Angel Ortega Núñez, MD, PhD:** Conceptualization, methodology, critical revision of the manuscript for important intellectual content, and supervision.

**Basilio José de la Torre Escuredo, MD, PhD:** Study design, data interpretation, writing – review and editing, and supervision.

## Funding

This study did not receive any specific grant from funding agencies in the public, commercial, or not-for-profit sectors.

## Abbreviations

AP: Anteroposterior
COR: Center of Rotation
SD: Standard deviation
SE: Standard error
HHS: Harris Hip Score
BMI: Body mass index
kg: Kilograms
PROMs: Patient-reported outcome measures
SF-12: Short Form Questionnaire - 12
WOMAC: Western Ontario McMaster Arthritis Index
